# Evaluating the shear bond strength and remineralization effect of calcium silicate‐based and conventional self‐adhesive resin cements to caries‐affected dentin

**DOI:** 10.1002/cre2.665

**Published:** 2022-10-03

**Authors:** Maryam S. Tavangar, Ayda Safarpour, Arefeh Torabi Parizi, Fereshteh Shafiei

**Affiliations:** ^1^ Department of Operative Dentistry, Oral and Dental Disease Research Center, School of Dentistry Shiraz University of Medical Sciences Shiraz Iran; ^2^ Students' Research Committee, School of Dentistry Shiraz University of Medical Sciences Shiraz Iran; ^3^ Department of Operative Dentistry, School of Dentistry Shiraz University of Medical Sciences Shiraz Iran

**Keywords:** calcium silicate, caries affected dentin, remineralization, self‐adhesive resin cement, shear bond strength

## Abstract

**Objective:**

Given the importance of preserving caries‐affected dentin (CAD) in conservative dentistry, the shear bond strength (SBS) of different resin cements to CAD has been investigated. Here, we aimed to compare the SBS and remineralizing effect of a calcium silicate (TheraCem) and conventional self‐adhesive cement (Panavia SA) on the SBS of CAD.

**Materials and Methods:**

Forty‐eight extracted third molars (24 sound and 24 CAD) were used. In each group, 12 teeth were prepared for bonding to TheraCem or Panavia SA. After removal of the enamel and caries, resin composite cylinders were luted on the prepared dentin. After 28 days of storage in the artificial saliva, SBS was measured and the failure mode analysis was investigated. The images of fractured sections were analyzed using scanning electron microscopy and energy‐dispersive X‐ray to evaluate the Ca/P weight ratio.

**Results:**

SBS of CAD and sound dentin was not different when cemented with TheraCem (9.56 ± 4.51 vs. 9.17 ± 2.76, *p* = .806), but the CAD showed significantly lower SBS to Panavia SA (9.4 ± 2.36 vs. 7.39 ± 2.18, *p* = .015). The Ca/P ratio in CAD was significantly higher when bonded to both TheraCem and Panavia‐SA than that of the controls (*p* = .001); however, this ratio was not different for those bonded to TheraCem compared to Panavia SA.

**Conclusions:**

Based on our results, TheraCem as a calcium silicate cement shows better SBS to attach the restoration to CAD as compared to Panavia SA. Obliteration and mineralization of the dentinal tubules in TheraCem were also higher than in Panavia SA. However, their ability to improve the amount of the Ca/P ratio in CAD was similar.

## 1 INTRODUCTION

Carious dentin consists of two layers. The outer layer known as caries‐infected dentin (CID) is highly demineralized, is physiologically not remineralizable, and contains irreversibly denatured collagen fibrils with the disappearance of cross‐linkages. On the other hand, the inner layer or caries‐affected dentin (CAD) is partially demineralized and physiologically remineralizable (Kuboki et al., 1977). High porosity, partially demineralized dentin, exposure of collagen fibers along with a decrease in the surface energy, and the reduction in intermolecular cross‐links of the collagen are the alterations shown in this tissue (Taniguchi et al., 2009). Meanwhile, there is evidence indicating that structural changes in CAD are reversible (Meraji et al., 2018). Consequently, minimally invasive restorative dentistry is recommended to preserve the CAD to protect the tooth structure and vitality of the pulp in direct and indirect restorations (Elsharkawy, 2018; Pintado‐Palomino et al., 2019). However, because of the chemical and mechanical alterations in CAD, such as a thicker and irregular smear layer with fibril‐like structures, and the presence of narrow and occlude dentinal tubules (Meraji et al., 2018; Pintado‐Palomino et al., 2019; Taniguchi et al., 2009), bonding to CAD is challenging. Several studies indicated that CAD showed lower bond strength compared to sound dentin (Elsharkawy, 2018; Meraji et al., 2018; Pintado‐Palomino et al., 2019).

The type of adhesive and cement is a determinant factor in the amount of bond strength to CAD (Palma‐Dibb et al., 2003). Currently, the use of bioactive materials such as glass ionomer cements, composite resins, and adhesives containing bioactive materials is recommended to restore CAD. These materials release ions (e.g., calcium and phosphate) that induce mineral deposition and remineralization of CAD and improve mechanical strength, thus extending the durability of the resin‐dentin bond (Elsharkawy, 2018; Alrahlah, 2018; Profeta et al., 2012, 2013; Sauro et al., 2012). In addition, these materials reduce biofilm penetration into the marginal gaps because of the toxic effect of fluoride, calcium, and phosphate ions.

Self‐adhesive resin cements are now very popular among dentists as they can provide a reliable bond to the dental structures while eliminating the etching procedure and need for bonding application (Mahrous et al., 2020). TheraCem (Bisco) and Panavia SA luting plus (Kuraray) are self‐adhesive resin cements containing methacryloxydecyl‐dihydrogen‐phosphate (MDP), as an acidic monomer, to provide superior hydrolytic stability. Panavia SA luting cements contain silane coupling agents to provide effective bonding ability, particularly to ceramic surfaces (Yoshihara et al., 2020). TheraCem, as a newly introduced calcium silicate cement, showed an ability to form crystalline calcium hydroxide, which may improve the mechanical bond between these cements and dentin (Meraji et al., 2018). It has the ability to release fluoride and calcium ions, contributing to the remineralization of the tooth mineral structures. Thus, it is suggested as a bioactive material (Van Landuyt et al., 2007; Mahrous et al., 2020; Yoshihara et al., 2010).

Given the importance of preserving CAD in conservative dentistry, the bond strength of different resin cements to CAD has been investigated. To the best of our knowledge, there are no studies to investigate the bond strength of TheraCem to CAD and its remineralization effect on this substrate. Accordingly, this study aimed to investigate the SBS strength of TheraCem to CAD in comparison with Panavia SA, as a conventional resin cement. Also, the mineral content of the bonded dentin would be evaluated as it is claimed that TheraCem can release calcium monophosphate ions. The null hypothesis of this study is that the remineralization effect and the bond strength of both conventional and Ca‐silicate‐based resin cements on sound and CAD are not different.

## 2 METHODS

### 2.1 Materials

Table 1 presents the main materials used in the study.

**Table 1:** Materials used in the study

Material	Type	Ingredients	Steps of application	Manufacturer
TheraCem	Self‐adhesive resin cement	Base: Calcium base filler, glass filler, dimethacrylates, ytterbium fluoride, initiator, amorphous silica	(1) Preparation procedure: – Clean the surface with water. Rinse thoroughly – Dry the surface using a strong stream of air for 3–5 s (2) Mixing procedure: – Dispense a small amount of material onto a mixing pad to eliminate any voids in each chamber of the dual syringe – Attach a mixing tip to the dual‐syringe – Pressing the plunger will mix and dispense the material (3) Cementation procedure: – Apply cement to the tooth preparation – Remove any excess cement (light cure the margins 2–3 s) – Final cure (light cure 20–30 s)	Bisco
		Catalyst: Glass filler, methacryloyloxydecyl‐dihydrogen‐phosphate (MDP), amorphous silica		
Panavia SA luting plus	Self‐adhesive resin cement	Paste A: MDP, Bisphenol A diglycidylmethacrylate (Bis‐GMA), triethyleneglycol dimethacrylate (TEGDMA), hydrophobic aromatic dimethacrylate, 2‐hydroxymethacrylate (HEMA), silanated barium glass filler, Silanated colloidal silica, dl‐camphorquinone, peroxide, catalysts, pigments	(1) Preparation procedure: – Clean the surface with water – Dry the surface (2) Mixing procedure: – Dispense an equal amount of paste A and B – Mix for 10 s (3) Cementation procedure: – Apply cement to the tooth preparation – Remove any excess cement (light cure the margins 2–5 s) – Final cure (light cure 10 s)	Kuraray
		Paste B: Hydrophobic aromatic dimethacrylate, hydrophobic aliphatic dimethacrylate, silanated barium glass filler, surface‐treated sodium fluoride, accelerators, pigments		

### 2.2 Specimen preparation

Forty‐eight extracted third molars, 24 sound and 24 with occlusal pit and fissure caries, that were extracted for definite clinical reasons (nonresearch purposes), such as caries, orthodontic purposes, and periodontal diseases, were used in this study. The teeth with cracks, severe caries, and previous treatment with chemical agents were excluded from the study.

The teeth were cleaned with fluoride‐free prophylaxis paste (Protec prophylaxis paste, Medicept) and then kept in 4% thymol solution (Sigma‐Aldrich) at room temperature for 2–3 weeks before the examination. The enamel of the occlusal surface was removed using a diamond disc (Drux) and then ground flat with 600‐grit silicon carbide abrasive paper (Extec Corp.) for 1 min under running water to create a uniform smear layer.

For the teeth affected by caries, the dentin was removed using a diamond disc (Drux) until the CAD remained. To detect the CAD, the exposed dentin was examined visually and tactility. Surfaces with discolored dentin and resistance to the excavator were considered CAD. This diagnosis was confirmed by caries detector solution (Kuraray Co.) applied on the dentin surface following the manufacturer's instructions by the second operator (restorative specialist). The teeth were ground until the bright pigmentation of the CID was removed, and the CAD remained. Then, the samples were mounted using polyethylene mold with self‐cured acrylic resin (Acropars, Marlik Co.) (Supporting Information 1).

### 2.3 Restorative procedures

In both sound and affected groups, the teeth were divided randomly into two groups (*n* = 12). One group was prepared for bonding to Theracem (Bisco) and the other one was for bonding to Panavia SA (Kuraray), according to the manufacturer's instructions. The occlusal surface of all samples was rinsed with distilled water and air‐dried gently before the application of the cement. Resin composite (FiltekTM Z250, 3M ESPE) cylinders were fabricated in the polyethylene mold with a dimension of 4 mm in diameter and 2 mm in length and polymerized using a light‐emitting diode (LED) unit (LED.F; Woodpecker) with a standard method. The blocks were luted on the prepared dentin with each cement. Subsequently, the excess resin cement around the composite block was removed carefully with a disposable brush, and the cement was photopolymerized with the LED light cure unit, according to the manufacturer's instructions (Table 1). While the cement was becoming polymerized, constant pressure was applied simultaneously as the static loading to the cementation mold for 5 min.

After performing the restorative procedure, due to the claimed ability of Ca‐silicate‐based cement (TheraCem) to release calcium monophosphate ions, all the tests including shear bond strength (SBS), evaluation of the mineral content of the bonded dentin using the energy‐dispersive X‐ray (EDX) method, and scanning electron microscopy (SEM) analysis were done after 4 weeks of aging in artificial saliva.

### 2.4 SBS

After the initial setting of cement according to the manufacturer's instructions, the molds were removed, and the samples were stored in artificial saliva (NikCeram Razi corp.) in an incubator (Cooled Incubator NUVE ES 250) at 37°C for 28 days, which was changed every day (Shaik et al., 2017). To avoid bias in data collection, we considered blinding during testing the specimens.

Shear bond testing was performed using a universal testing machine (Instron, Z020; Zwick Roell). The samples were placed in a jig, and the force was applied to each specimen parallel to the bonded interface at a crosshead speed of 1 mm/min until failure occurred. The SBS was calculated using the software TestXpert II (Zwick Roell).

### 2.5 Failure mode analysis

The debonded surface of the samples was evaluated for failure mode analysis under ×10 magnification and categorized into three modes:

Mode 1: Adhesive failure that occurred at the filling material and dentine interface

Mode 2: Cohesive failure within the filling material

Mode 3: Mixed failure mode (both adhesive and cohesive failures).

### 2.6 SEM

The images of the fractured sections of three samples from each group were examined using an SEM (TESCAN‐Vega 3; TESCAN) at 15 kV and with magnifications of ×500 and ×3500. To prepare the samples, we dried them in a desiccator and vacuum‐coated them with approximately 10 nm thickness of gold.

### 2.7 EDX analysis

Seven samples of each group were sectioned perpendicularly to the bonded interface using a diamond disc (Drux), as near as possible to the center of the cylinder. Furthermore, two groups of uncemented sound and CAD as the control groups were also sectioned. Then, all the sections were polished with abrasive discs (Extec Corp.), cleaned with distilled water, and dried after each polishing step. These samples were prepared similarly to those used for the SEM and examined with the same machine equipped with an EDX spectrometer at 20 kV. The mineral content of intertubular space, just under the restoration–dentin interface, was measured.

### 2.8 Statistical analysis

Data are represented as mean ± SD. SPSS software version 22 (SPSS Inc.) was used to analyze the data using an Independent sample *t*‐test for comparisons between the cements or dentin substrate. Also, one‐way analysis of variance was used for the comparison of EDX in the three groups. The values lower than .05 were considered statistically significant.

## 3 RESULTS

### 3.1 SBS

**Figure 1:**
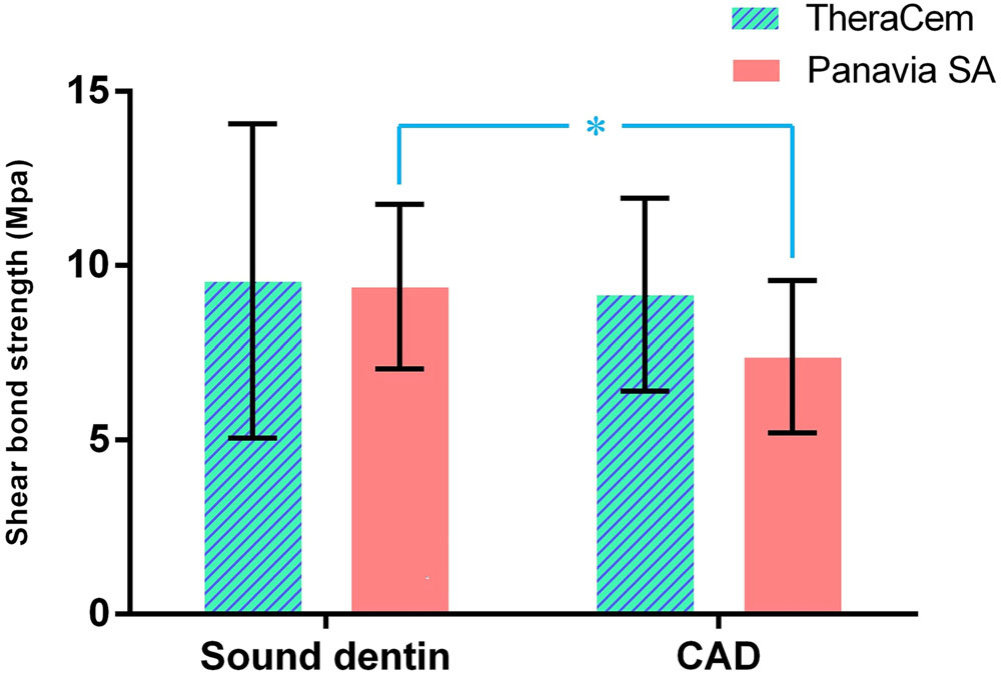
Shear bond strengths of different groups (MPa). The groups with a significant difference are marked with an asterisk.

A comparison of SBS of sound and CAD to Panavia SA and TheraCem showed no difference (*p* = .99 and .47 to sound and CAD, respectively). While for TheraCem‐bonded specimens, there was no significant difference between the two groups of sound dentin and CAD (*p* = .806), in Panavia SA‐bonded specimens, the CAD groups showed significantly lower bond strength compared to sound dentin (*p* = .015, Figure 1).

### 3.2 Failure mode analysis

The modes of failure distributions are represented in Table 2. In all groups, the frequent failure mode was a mixed failure. The cohesive failure was only recorded for sound dentin specimens bonded to TheraCem. The most adhesive failure was found for CAD bonded to Panavia SA (Supporting Information: Figure 2).

**Table 2:** Specimen numbers of different failure modes for each group

Material	Dentin substrate	Failure mode		
		Cohesive	Mixed	Adhesive
TheraCem	Caries affected dentin	0	9	3
	Sound dentin	2	9	1
Panavia SA luting plus	Caries affected dentin	0	7	5
	Sound dentin	0	10	2

### 3.3 SEM

In the SEM evaluations in lower magnifications, in the CAD samples that were bonded to Panavia SA, the dentin surfaces showed small areas of covering with few islands on the surface. However, TheraCem spread more homogenously over the CAD surface and with more islands of cement distributed over the remaining area.

**Figure 2:**
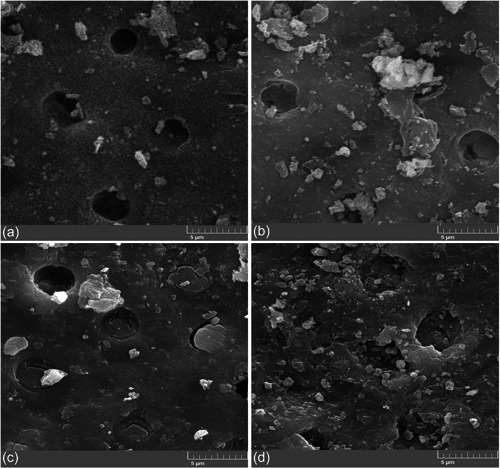
Scanning electron microscope examinations: (a) sound dentin bonded to the Panavia SA. (b) Caries affected dentin (CAD) to the Panavia SA. (c) Sound dentin bonded to the TheraCem. (d) CAD bonded to TheraCem.

SEM images revealed depositions on the treated dentinal tubule openings in all groups. Complete obliteration and deposition of minerals in dentinal tubule opening in the samples bonded to TheraCem were more frequently observed than those bonded to Panavia SA, particularly in the CAD surface. Figure 2 shows the results of SEM imaging.

### 3.4 EDX analysis

Table 3 displays the means and standard deviations for the calcium/phosphate weight ratio of SD and CAD bonded to both TheraCem and Panavia SA resin cements and two groups without any cementation as the positive and negative control.

**Table 3:** Average of the Ca/P weight ratio

Materials	Substrate		
	Sound dentin	Caries affected dentin	*p* Value
TheraCem	2.39 ± 0.03^A^	2.28 ± 0.29^C^	.29
Panavia SA luting plus	2.22 ± 0.16^B^	2.19 ± 0.19^C^	.67
Control	2.40 ± 0.10^A^	1.60 ± 0.06^D^	.001
P value	0.007	0.001	

*Note*: Data are represented as mean ± SD. In each column, similar superscript letters indicate no significant difference between the two items.

In CAD, EDX analysis showed that the Ca/P weight ratio was significantly higher when bonded to both Panavia SA and TheraCem than that of negative control (*p* = .001). The mineral content of CAD samples bonded to TheraCem was not significantly different from those bonded to Panavia SA.

**Figure 3:**
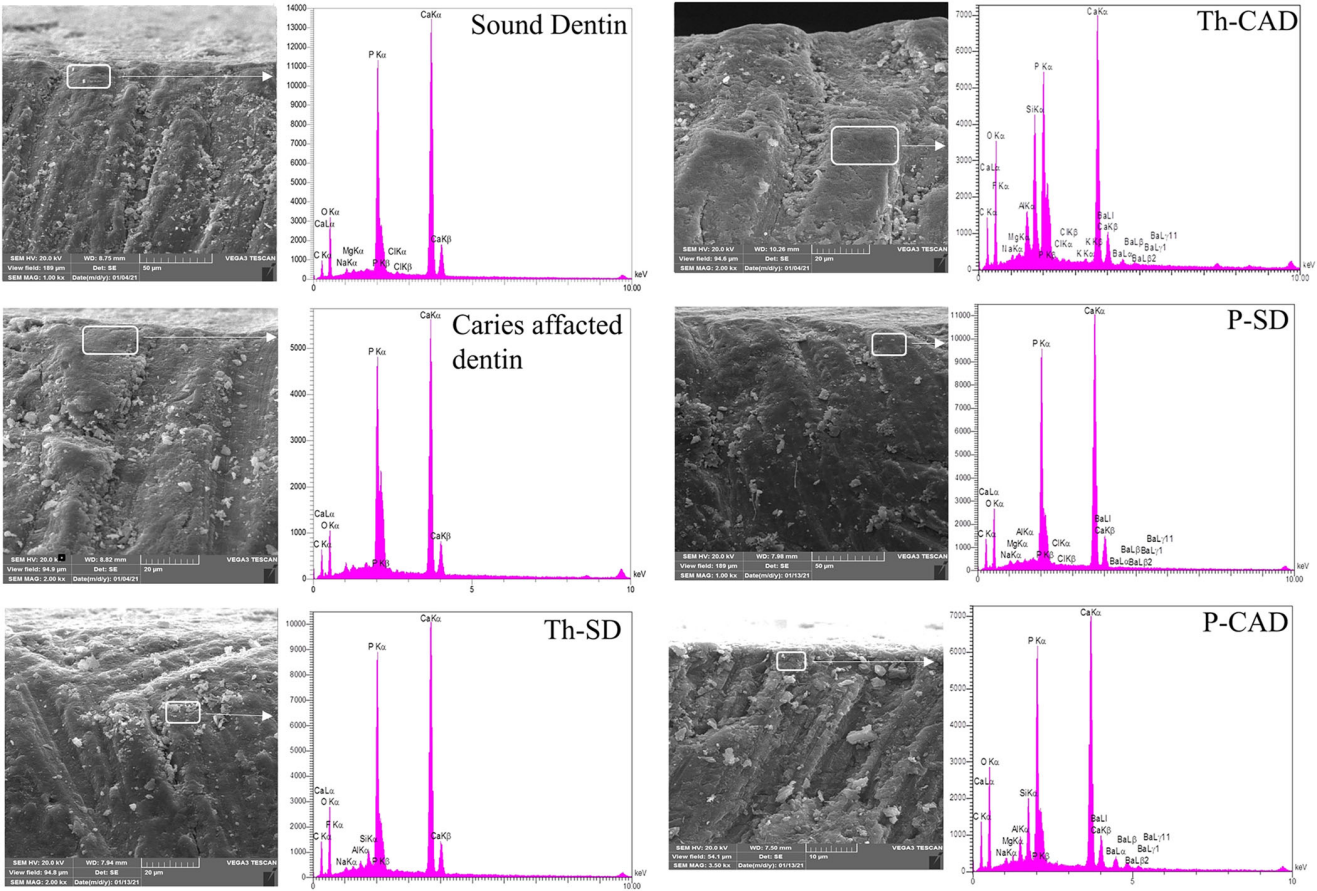
Energy dispersive spectroscopy plots of the control group and the test materials accompanied by SEM micrographs of the intertubular space at the site where the EDX analysis was performed are marked by rectangles. CAD, caries affected dentin; EDX, energy‐dispersive X‐ray; P, Panavia, SEM, scanning electron microscope; SD, sound dentin; Th, TheraCem.

Energy dispersive spectroscopic plots of two test materials and the control group in sound and CAD are shown in Figure 3. In SEM images, the intertubular area, which was investigated by EDX analysis, was defined (Figure 3). The EDX analysis for all groups (TheraCem, Panavia SA, control) showed strong peaks of calcium and phosphate. In addition, the highest peak of oxygen and silica among all groups was noticed for CAD specimens bonded to TheraCem. Furthermore, CAD specimens bonded to TheraCem showed a stronger peak of aluminum than all other groups.

## 4 DISCUSSION

In the present study, we evaluated the SBS of a calcium silicate‐based resin cement (TheraCem) and the conventional self‐adhesive resin cement (Panavia SA) to the sound and CAD. Here, we noticed that TheraCem shows a similar SBS to both CAD and sound dentine, while Panavia SA cannot. It also shows a less adhesive failure mode. Consequently, TheraCem may be more effective for restorations on CAD.

The results from previous studies indicate that the SBS of various types of cement to CAD and sound may be dependent on the material. Suzuki et al. in an investigation on etch and rinse (RelyX ARC), self‐etch (Panavia F), and self‐adhesive (RelyX Unicem) resin cements showed that there was not a significant difference between the microtensile bond strength of these cements to CAD and sound dentin in each group (Suzuki et al., 2013). In another study, Czarnecka et al. showed that the SBS of automixed GIC to the sound dentin was not significantly different from CAD, while the SBS of the hand‐mixed GIC to the sound dentin was higher than CAD (Czarnecka et al., 2007). Choi et al. reported that the microtensile bond strengths of conventional GIC and RMGIC to CAD were significantly lower than those of the sound dentin (Choi et al., 2006). Our findings indicating that the SBS of TheraCem to the sound and CAD is similar and that for Panavia SA is lower to CAD provide another evidence that the SBS of some materials depends on their biochemical properties.

The results from the present study, depicting a similar SBS to sound dentin for both TheraCem and Panavia SA cement, may be explained by the presence of the same functional monomer (MDP), which makes their stable bond to dentin surfaces. It is established that the adhesion of resin cement to teeth depends on their micromechanical interlocking and chemical adhesion to the tooth substrate (Mahrous et al., 2020). It was reported that the type of the functional monomer in adhesives may play a major role in providing a durable chemical bond to hydroxyapatite (Yaguchi., 2017). In addition to the acidic monomers, there are other components in TheraCem that are key to its bonding capability. Active ingredients such as calcium and phosphate incorporated in this cement can ionically interact with hydroxyapatite (Piwowarczyk et al., 2004). There are some evidence that showed that adhesive resins with added calcium and phosphate ions can act as an ion reservoir that facilitates the remineralization of the tooth surfaces and may enhance the SBS to tooth structures (Ranjkesh et al., 2016; Shadman et al., 2015). Some previous studies have clarified that CAD may exhibit a lower bond strength to the cements and this has contributed to the partial demineralization in this tissue (Meraji et al., 2018). Consequently, the remineralization effect of TheraCem may explain its better SBS to CAD as compared to Panavia SA.

In our study, SEM images showed obliteration and mineralization of the dentinal tubules in TheraCem, which were more frequent than in Panavia SA. In addition to the probability of mineral deposition in the dentinal tubules, more alkaline properties of these cements, compared to other self‐adhesive cements, may explain less dissolution of the smear layer and probably keep more smear plugs within the opened dentinal tubules (Miletic, 2018; Yaguchi, 2017).

In this study, the mixed failure mode was predominant in all groups. The cohesive failure was only recorded for sound dentin bonded to TheraCem, which may be related to the low resistance of the tested material itself rather than its true bond strength to the dentin (Bonifácio et al., 2012; Choi et al., 2006). Also, it may be related to numerous porosities in the structure of the material, which may act as stress points (Hoshika et al., 2015). In addition, the most adhesive failure was found for CAD bonded to Panavia SA. Adhesive failures in CAD may be related to several factors like the presence of collagen fibrils partially denatured by bacterial acids and/or metalloproteinases (Kuboki et al., 1977; Pashley et al., 2004) and an incomplete infiltration of the luting agent into the demineralized dentin that is commonly related to these failures (Hashimoto et al., 2002).

Here, we noticed that despite the finding that the Ca/P weight ratio was higher in the CAD group bonded to both TheraCem and Panavia SA compared to unbonded CAD, there was no significant difference between the two types of cement. It is well known that calcium silicate cements, under a wet condition, can release calcium and form apatite crystals. They can also form calcium hydroxide, which is a highly alkaline material and, hence, may cause degradation of the exposed demineralized collagen (Camilleri, 2014; Chen et al., 2018; Huang et al., 2020). Although calcium silicate cements may deliver the minerals to the demineralized dentin and may increase its hardness, it is shown that they cannot induce intrafibrillar remineralization and recovery of a sound modulus of elasticity (Schwendicke et al., 2019). The observed peaks of ions other than calcium and phosphate may be related to the main component of the cement, also the mineral content dissolved by the functional acidic monomer, and the filler particles released from the cement itself (AL‐Kataan and Ali 2021).

It should be noticed that our study, as an in‐vitro study, had some limitations. As the SBS test must be performed on various specimens, the variability among the test groups could not be avoidable. Thus, the next studies are suggested using the microtensile test, which allows multiple specimens of carious and sound dentin obtained from the same tooth to ensure an accurate comparison. Furthermore, having a longer storage time and performing hardness and XRD tests would be beneficial for the evaluation of CAD after cementation by Ca silicate cements.

## 5 CONCLUSION

Based on our findings, it is concluded that TheraCem, as a calcium silicate cement, shows better SBS to attach the restoration to CAD as compared to Panavia SA. Obliteration and mineralization of the dentinal tubules in TheraCem were also higher than in Panavia SA. However, their ability to improve the amount of the Ca/P ratio in CAD is similar. Based on our findings, it may be suggested that calcium silicate cement has a remineralization effect in addition to improving bonding strength to CAD.

## AUTHOR CONTRIBUTIONS

All authors contributed to the conception, design, data acquisition and interpretation, and statistical analysis, drafted, and critically revised the manuscript. All authors gave their final approval and agree to be accountable for all aspects of the work.

## CONFLICT OF INTEREST

There is no conflict of interest.

### ETHICS STATEMENT

This study was approved by the Research Ethics Committee of Shiraz University of Medical Sciences (Ethical Approval Number: IR.SUMS.DENTAL.REC.1399.191) and conforms with the declaration of Helsinki and follows the protection of human subjects' guidelines. From all participants, informed written consent was taken.

## Supporting information

Supporting information.

## Data Availability

The data that support the findings of this study are available from the corresponding author upon reasonable request.
