# Supplementary material for: Evaluating the shear bond strength and remineralization effect of calcium silicate‐based and conventional self‐adhesive resin cements to caries‐affected dentin
Source: Clin Exp Dent Res. 2022 Oct 3;8(6):1630–7. doi: 10.1002/cre2.665 (PMC9760150; doi:10.1002/cre2.665)
Supplement: Supplementary file 1 — Supporting information. [file CRE2-8-1630-s001.docx]

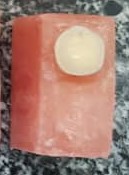

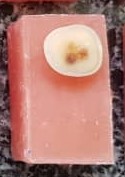


**Online supplement1 :** Specimens with exposed surface of CAD and sound dentin mounted in acrylic resin

**
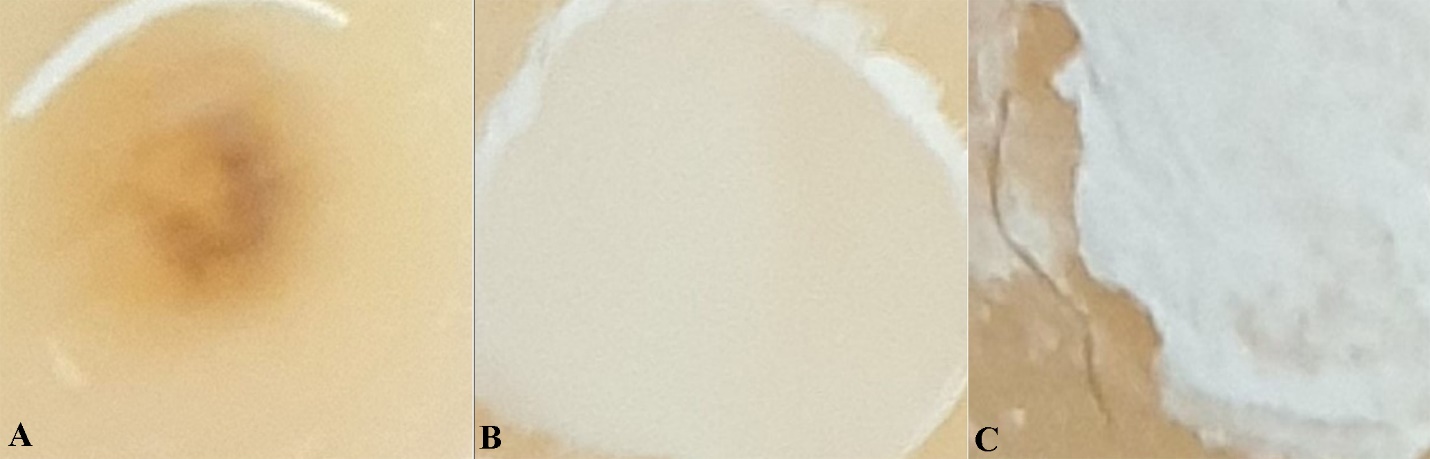
**

**Online supplement 2: Failure mode A) Adhesive failure mode, B) Cohesive failure mode, C) Mixed failure mode**
